# Epidemiology of playground equipment related/unrelated injuries to children: A registry-based cohort study from 6 emergency departments in Korea

**DOI:** 10.1097/MD.0000000000013705

**Published:** 2018-12-14

**Authors:** Dongbum Suh, Jin Hee Jung, Ikwan Chang, Jin Hee Lee, Jae Yun Jung, Young Ho Kwak, Do Kyun Kim

**Affiliations:** a Department of Emergency Medicine, Seoul National University Hospital, Seongnam, Gyeonggi-do; b Department of Emergency Medicine, Seoul National University Boramae Hospital, Seoul; c Kangwon National University College of Medicine, Chuncheon, Gangwon-do; d Department of Emergency Medicine, Seoul National University Hospital, Seoul; e Department of Emergency Medicine, Seoul National University College of Medicine, Seoul.

**Keywords:** emergency department, equipment, injury, playground

## Abstract

The aim of study was to understand the epidemiology of playground injury and to find the factors related to the clinically significant injuries. This retrospective observational study enrolled children (age 0–18 years old) who visited the emergency departments (ED) of 6 hospitals in Korea.

We obtained and analyzed the data from the ED injury surveillance system, which was supported by the Korea Centers for Disease Control. Clinically significant injury (Cs injury) was defined as the injuries that caused hospital admission for more than one day. The factors associated with injury and clinical outcome were compared between admitted and discharged patient groups. Multivariable logistic regression and the population attributable fraction were used to identify significant factors for hospitalization.

A total of 1458 patients were enrolled. The proportion of patients who visited ED due to injuries unrelated to the playground equipment use was 57.8%. The majority of Cs injury was upper extremity fractures (68.1%). The risk factors for admission were the 6- to 11-year old age group (OR 5.7, 95% CI 1.3–25.0) and public playground (OR 2.4, 95% CI 1.1–5.3); the population attributable factor of these factors was 51.3% and 36.0%, respectively.

This study shows that approximately 60% of the patients visited ED due to injury unrelated to the playground equipment use. The risk factors of Cs injuries were ages 6 to 11 and public playgrounds. The results of the study can be helpful to formulate the prevention policy against playground injury.

## 1 Introduction

Outside play activity for children is an essential activity that has immense benefits not only for developing motor and cognitive functions of children but also for developing their social skills and interpersonal relationships.^[1]^ The playground is the representative space where such gatherings and outside play activities occur. However, these otherwise beneficial activities are also accompanied by risk of injury.^[2]^ According to data from the National Electronic Injury Surveillance System (NEISS), a basic data collection system of injuries in the United States, approximately 200,000 children per year visit emergency departments (ED) as a result of playground equipment-associated injuries. The severity of the injuries in the above statistics comprises minor injuries, including abrasions, and severe injuries, including death. Approximately 3 to 6% of the ED admission patients require inpatient treatment.^[3–5]^

Epidemiological studies using injury data primarily from the United States and Canada were conducted to investigate the magnitude and characteristics of children's playground injuries and formulate a preventive policy.^[3,4,6]^ According to the preceding studies, children who had used medical facilities due to playground-associated injuries were most often between 5 and 9 years old. Most children also suffered an injury due to a fall and damaged their upper limbs. Furthermore, the injuries have been reported to frequently occur from playground equipment near schools. In particular, children are injured while using climbing bars or swings. Another preceding study reported that the underlayment of the playground is also associated with playground injuries of children.^[7–9]^

However, the majority of such epidemiological studies have been conducted using data limited to the United States and Canada.^[10]^ Furthermore, the data solely analyzed patients particularly injured by playground equipment.^[2–8]^ As such, the aforementioned studies did not include the myriad types of injuries that can occur within the playground, apart from the use of playground equipment. In addition, as the majority of the patients in the epidemiological studies only had suffered minor injuries, the preceding studies did not accurately reflect the epidemiological data of severe injuries, which require large resource expenditures. When regarding injury prevention policies for playgrounds, it is important not only to collect data from the region in question but also to gather and analyse data of playground injuries unrelated to the use of playground equipment that takes up a significant portion. Therefore, a study that is centered on patients with severe clinically meaningful injuries can be more helpful to formulate comprehensive and effective injury prevention methods.

The Korea Center for Disease Control (KCDC) has established an integrated injury surveillance system to investigate injury incidence statistics as well as preventive factors.^[11]^ Among various injury surveillance system components, the in-depth injury surveillance survey of ED patients designates data collection of patient injuries regarding the injury mechanism, cause, and related factors of admission to sample hospital EDs. In these data, the site of injury and the object that caused the injury can be distinguished clearly, such that ED patients admitted due to injuries associated with playground equipment and injuries associated with playground use in general can be categorized separately.

The aim of the present study is to investigate the aspects of clinically important playground injuries that required inpatient treatment with utilization of injury data from South Korea. In addition, factors related to such severe playground injuries were also examined. Results would be valuable to assist in policy formulation of playground injury prevention and resource distribution.

## 2 Methods

### 2.1 Study participants

We selected 6 hospitals among medical institutions that were registered to the ED injury surveillance system that also permitted direct access of medical records to researchers. Among the selected hospitals, 5 hospitals were located at Seoul metropolitan city and Gyunggi Province, where approximately 43% of South Korean children (under the age of 18) reside. The distance of the hospitals from one another demonstrated an appropriate level of hospital distribution. The one remaining hospital is a major referral hospital in a rural area in southern part of the country (Gyeongsangnam Province). The participants of the study were patients under the age of 18 who visited the EDs due to injuries that occurred on a playground from January 1, 2011 to December 31, 2011. Injuries within the playground setting all were included in the study, even if the injury was unrelated to the use of playground equipment.

### 2.2 Data collection

The medical records of the patients were reviewed to obtain age, gender, date of injury, date of admission to the ED, injury type, location of playground, object/equipment that caused the injury, clinical diagnosis and affected body parts.

The collected data were categorized in the following manner: Age referred to the age of the patient at the time of admission to the ED. Injury type was categorized as falling, tripping, jams, impact, laceration, sprain, and others. The location of playground was categorized into residence playgrounds (i.e., playground in an apartment complex, close to the home of the victims), playgrounds in an education institution (i.e., school or preschool playground), public playgrounds (i.e., playground in a park, public playgrounds that do not require an entrance fee), and others. The objects/equipment that caused the injury was categorized into playground equipment, playground structures, and other. We define playground equipment as recreational equipment designed for children to ride on for fun, and playground structures as structures in a playground not for play, but for convenience or decoration such as benches, fence, trees, bushes, and sculptures. Playground equipment included slides, swings, pull-up bars, jungle gyms, climbing rides, crossing rides, spinning rides, shaking rides, air rides, and others. Diagnoses and affected body parts were categorized based on the international statistical classification of diseases and related health problems (ICD)-10; diagnoses were categorized as contusions or abrasions, strains and sprains, lacerations, fractures, concussion, internal organ injuries, and others. Affected body parts were categorized as head, face, neck, body, shoulder/arm, butt/legs, and others. When multiple injuries occurred, we referred to medical records to select the injury with the highest Abbreviated Injury Scale (AIS) value.

### 2.3 Statistical analysis

STATA 11.2 (Stata Corporation, East College Station, TX) was used for statistical analysis. Discrete variables were expressed as a percentage and a frequency. The differences between the 2 groups were analysed using Pearson's chi-square test. Continuous variables were expressed using the mean and standard deviation. Multivariate logistic regression analysis was performed to compute the odds ratio and 95% confidence intervals. Odds ratios were expressed in 2ways: the odds ratio after adjusting for age and gender and the odds ratio after adjusting for all variables. The STATA command “punaf” was used to calculate the population attributable fraction (PAF) of the investigated factors with respect to the fractures associated with playground rides. In the present study, PAF is a fractional expression of the intraplayground injury risk factor over the injury incidence that required inpatient treatment. The significance level was set at *P* < 0.05.

### 2.4 Ethics statement

The study protocol was approved by the Institutional Review Board of Seoul National University Hospital (IRB No. H-1407-047-593). Informed consent was waived by the board.

## 3 Results

### 3.1 The overall characteristics of patients

**Figure 1:**
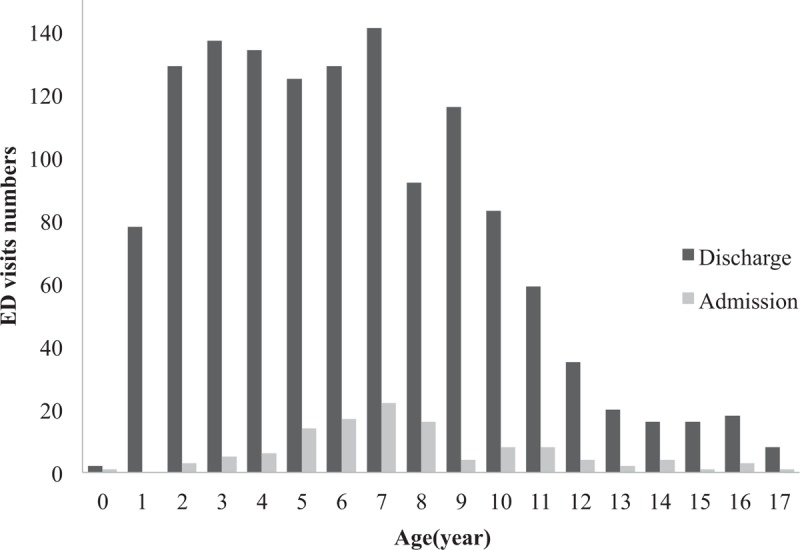
Age of victims of injury in playground by disposition.

**Figure 2:**
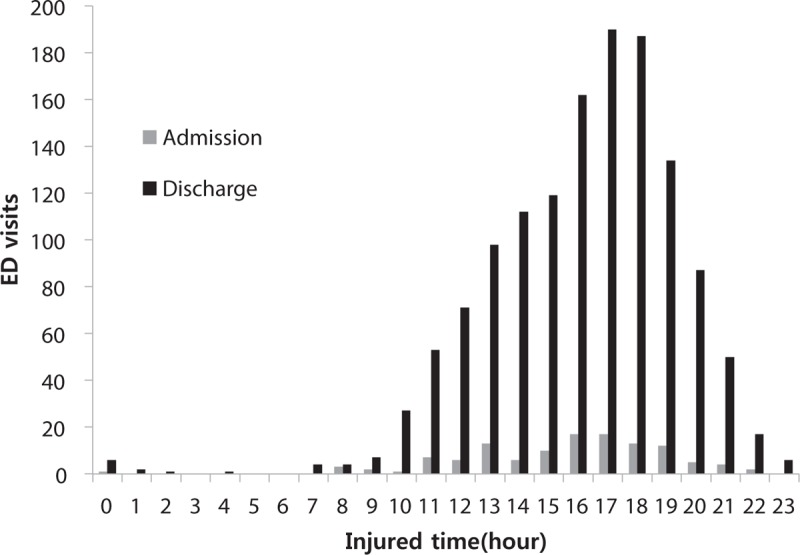
Injured time of injuries in playground by disposition.

A total of 1458 patients under the age of 18 visited the EDs during the study period due to injury at playground. Among the injured patients, 119 patients (8.2%) were hospitalized for inpatient treatment. The average age of the injured patient was 6.5 (± 3.6). When the age distribution was examined in detail, 3 injured patients were infants under 12 months (0.2%), and relatively similar distribution was found from 2 to 7 years old. After the age of 10, the frequency of injury dramatically decreased and only 8.8% of the injured patients were over the age of 12 (Fig. 1). The distribution of the time of injury revealed that the frequency of injuries increased after 9 am when patients begin their daily activities. The most frequent time of injury was between 4 and 6 pm (Fig. 2).

Injuries related to the use of playground equipment constituted 42.2% of the total number of playground injuries. Injuries of more than half of the patients (52.0%) occurred at public playgrounds, followed by resident playgrounds (31.6%) and school playgrounds (15.0%). The most common injury type was impacts (37.0%), followed by falls (29.8%) and trips (18.5%). More than half of the injured patients injured their head/face/neck area (55.8%), followed by upper limb injuries (24.1%). The most common injury was laceration of the head/face/neck with 356 patients (24.4%) afflicted, followed by contusion of the head/face/neck (216 patients; 14.8%) and upper extremity fractures (209 patients; 14.3%) (Table 1).

**Table 1:**
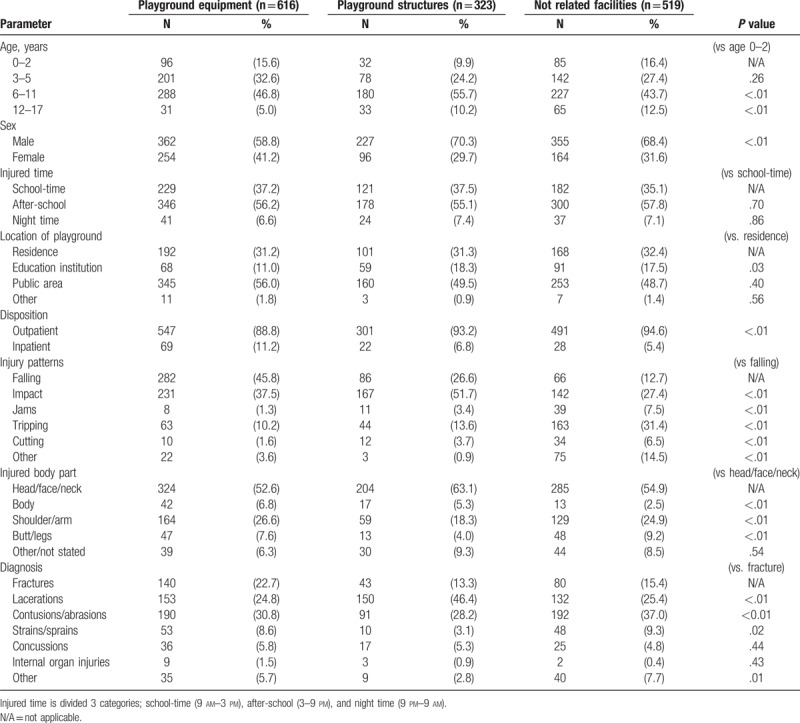
Difference of playground injury patterns by injury-related equipment.

### 3.2 Analysis of playground injuries unrelated to playground equipment use

Playground injuries unrelated to playground equipment use accounted for 842 cases (57.8%). Among these, there were 323 cases due to playground structures (22.2%) and 519 (35.6%) injuries unrelated to the facility. The average age of the patients injured from playground equipment (ride) use was 6.1 (± 3.4) while the average age of the patients injured from playground structures was 7.1 (± 3.4). The average age of patients with injuries unrelated to the facilities within the playground was 6.7 (± 3.9). The number of male patients was higher in the group with injuries unrelated to playground rides than the group with injuries from the use of playground rides (58.8% vs 69.1%, *P < *.001).

School playgrounds exhibited relatively higher incidences of injuries unrelated to playground equipment use compared to those of injuries associated with playground equipment use (11.0% vs. 17.8%, *P < *.001). Injuries associated with playground structures commonly resulted in higher incidences of head/face/neck injuries compared to injuries due to other reasons. In addition, injuries associated with playground structures were typically diagnosed as lacerations. Injuries associated with playground rides (equipment) consisted of head/face/neck lacerations (132 patients, 21.4%), upper extremity fractures (114 patients, 18.5%), and head/face/neck contusions/abrasions (110 patients, 17.9%). On the other hand, head /face/neck laceration accounted for 24.7% (208/842), head/face/neck contusions/abrasions 20.8% (175/842), and upper extremity fractures 11.3% (95/842) in the injured patients unrelated to playground equipment use.

### 3.3 Characteristics of clinically significant injuries

A total of 119 patients suffered from clinically significant injuries (Cs injuries) that required hospitalization for more than one day. The numbers of hospitalized patients exhibited a gradual increase until 7 years old, and after 7 years old, the numbers of hospitalized patients gradually decreased (Fig. 1). Among the hospitalized patients, 1.9% were 0 to 2 years old, 5.9% were 3 to 5 years old, 10.8% were 6 to 11 years old, and 11.6% were 12 to 17 years old. Injuries that required hospitalizations predominantly occurred approximately 1 pm and from 3 to 7 pm However, the injured patient to hospitalization ratio was the highest from 8 to 9 am (Fig. 2).

Among hospitalized patients, 42.0% of the injuries were unrelated to playground equipment. Playground location and hospitalizations also exhibited a meaningful relationship. While approximately 5.6% of the injured patients on resident playgrounds were hospitalized for inpatient treatment, school playgrounds and public playgrounds exhibited 10.1% and 9.4% hospitalization rates, respectively (Table 2).

**Table 2:**
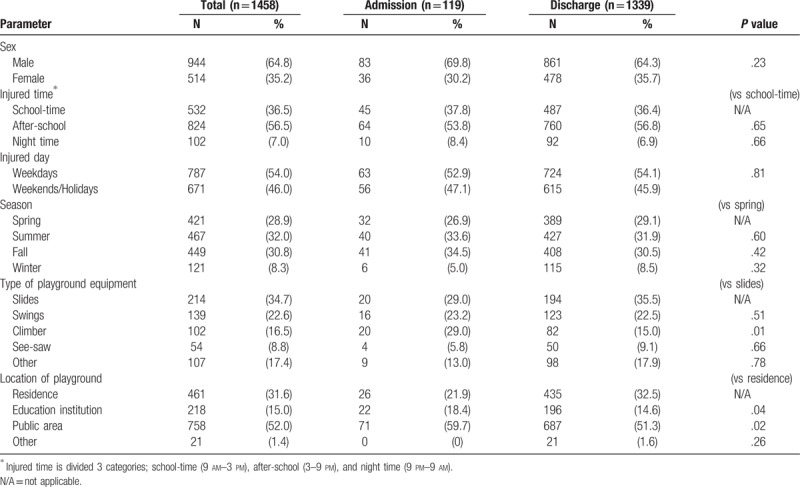
Pre-event factors of playground injuries by disposition.

Falls (63.0%) were the most common injury type of hospitalized patients, followed by impacts (16.8%) and trips (14.3%). Most of the injuries affected upper extremities as the primary site of injury (70.6%). Among the patients who needed hospitalization, the most common injury was upper extremity fracture (81/119, 68.1%), followed by lower extremity fracture (7/119, 5.9%) (Table 3).

**Table 3:**
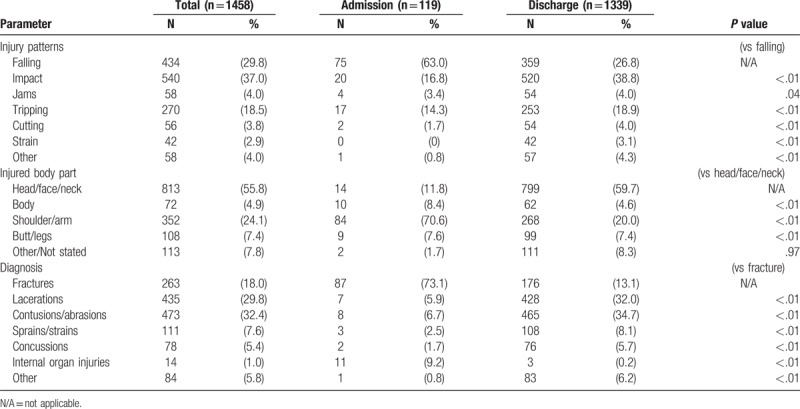
Injury patterns and clinical outcomes of the visiting of playground injuries by disposition.

### 3.4 Upper extremity fracture risk factors and population attributable fraction (PAF)

Upper extremity fractures, which comprised the majority of chief complaints (72.5%) for hospitalization among patients injured due to playground equipment use, were analysed in terms of hospitalization risk factors. Ages 6 to 11 (OR 5.7; CI 1.3–25.0) and public playgrounds (OR 2.4; CI 1.1–5.3) were identified as significant risk factors. The population attributable fractions of the 2 risk factors were 51.3% (CI 20.5–70.1) and 36.0% (CI 0.1–59.0), respectively (Table 4).

**Table 4:**
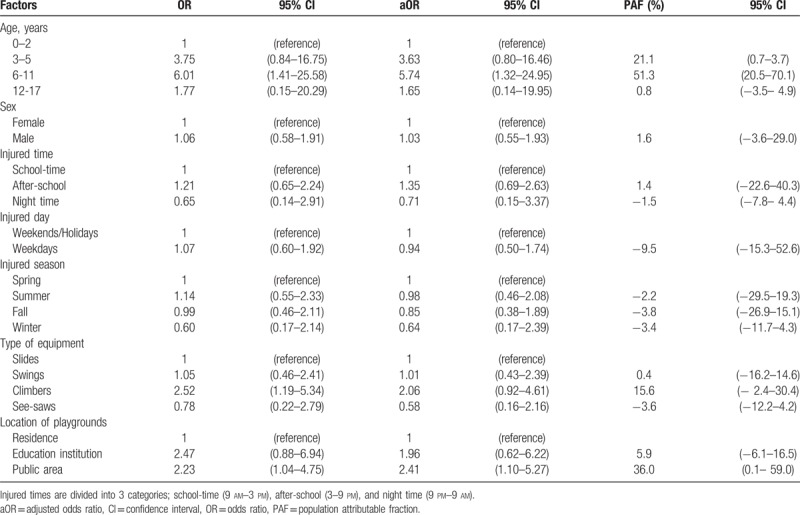
Population attributable fraction for admission due to upper extremity fracture based on OR for pre-event factors of playground equipment related injuries.

## 4 Discussion

Epidemiological studies on playground injuries, and more specifically, injuries associated with playground equipment, primarily are conducted with injury data from the United States and Canada.^[10]^ The NEISS is used to collecting ED admission data associated with injuries due to products in the United States.^[3–6]^ Likewise, Canada also collects hospital data associated with injuries through the Canadian Hospitals Injury Reporting and Prevention Program (CHIRPP).^[12–14]^ In South Korea, an in-depth injury surveillance system for EDs was established in 2006. The system compiles data from EDs of 20 hospitals around the country regarding the date of injury of the admitted patient, location of the injury, injury cause, injury type, and results. Unlike the data of other countries, these particular data clearly indicate whether the location of the accident was in fact a playground or not. As such, the data can distinguish whether the playground injury was related to the use of playground equipment or unrelated to the use of playground equipment. The present study is the first epidemiological study that used the aforementioned South Korean data to not only identify injuries due to playground equipment use but also identify injuries unrelated to playground equipment use.

According to a previous study in North America, injuries from playground equipment use were common between ages 5 through 8.^[15]^ In particular, injuries associated with climbing equipment, swings, and slides were common. The most common injury type was falling, and the most common affected body part was the upper extremities. Approximately 3% to 9% of all patients injured from playground equipment use have required inpatient treatment. The majority of the hospitalized patients exhibited a chief complaint of a fracture from falling.^[1–3,5]^ In the present study, the number of injuries from swings and slides was greater than that from climbing equipment. Furthermore, the most common injury type was impacts, and head and neck injuries were more common than upper extremity injuries. These results were somewhat different from those shown in previous studies.^[15]^ However, hospitalized inpatients constituted approximately 8% of the total number of patients with playground injuries. Hospitalizations were the most common between 5 and 8 years old, and climbing equipment, slides, and swings were all equally potentially dangerous. The majority of the hospitalized patients had fractures due to falling. Upper extremity injury was also common among hospitalized patients. The epidemiologic characteristics and results of the inpatients in this study were similar to those of the previous studies.

In the case of other countries, it is reported that injuries sustained in residential playgrounds were more severe and the hospitalization rate was higher than those in public playgrounds.^[3,16]^ Contrary to the above findings, however, the present study found that injuries such as upper extremity injuries sustained in public playgrounds required a greater number of hospitalizations. In particular, there were more public playground cases where incidences of fractures occurred due to a fall (56.3% vs. 80.4%, *P* = .04). Such differences may be attributed to the fact that the primary form of residence near the participating hospitals was apartments. As apartment playgrounds are well managed and regulated according to a safety standard designated by law, it is expected that residential playgrounds in Korea have lower incidences of injury than in other countries. Furthermore, in the case of public playgrounds, it is expected that more children will be using the facility simultaneously compared to a residential playground. Due to the ensuing congestion of the public playground, it is suspected that the public playgrounds in Korea had a higher risk of falling than a school or residential playground.

Of the inpatients, 68.1% were hospitalized due to upper extremity fracture. Ages 6–11 and playgrounds located in public places (public playgrounds) were important risk factors for hospitalization. The PAFs of the 2 risk factors were 51% and 31%, respectively. It means that if we keep children aged 6 to 11 from playing in the playground or if we eliminate all public playgrounds, the hospitalization due to playground injuries may be significantly prevented. But realistically we cannot make 6–11 ages not to play in the playground and also cannot remove all public playgrounds. Consequently, it is expected that emphasizing age and playground location-centred safety polices and education should help greatly in preventing playground injuries of children.

Unlike other studies, the present study comprehensively analyzed all injuries that occurred within the playground, including injuries not associated with playground equipment use. Children tend to engage in outdoor play activities by not only using the playground equipment but also using the rest of the playground structures. In our study, approximately 40% of the patients had been hospitalized due to an injury unrelated to playground equipment use. Currently, there are no clear studies to investigate this phenomenon. While there are laws regulating the playground equipment within the playground in South Korea, there are no safety measures regarding the rest of the playground structures. Sculptures and convenience facilities recently have been installed within the playground for aesthetic purposes. However, there are no policies that regulate such structures. Consequently, if there are many accidents caused by such structures, a safety regulation should be implemented in response.

A limitation of the present study is that we could not use the national statistics in their entirety and only compiled data from 6 selected hospitals from certain regions. As such, the present study suffers from a problem of representativeness. However, the locations of the selected hospitals are in areas where 43% of South Koreans under the age of 18 reside. We also believe that the results of the present study are relatively reliable as they are very similar to those of previous studies. In addition, injured patients who had been treated for outpatient clinic were not included in the study. This exclusion may have led to the underestimation of the actual injured population. Finally, we chose only one injury diagnosis with the highest AIS value in multiple body injuries for one case. Therefore, some significant injuries may be neglected if the patient had a more serious injury.

## 5 Conclusion

In conclusion, we showed the epidemiology of playground injury in Korea and found that approximately 60% of the patients visiting ED and 40% of the inpatients were injured patients unrelated to the playground equipment use. Upper extremity injuries accounted for a considerable number of hospitalizations and the risk factors were ages 6 to 11 and public playgrounds. Consequently, parents should be aware of these risk factors and oversee children's safety carefully for 6–11 aged during playing on the playground equipment and structure. And, playground safety education should be emphasized for the above age group, and a policy change is necessary to decrease the number of accidents within public playgrounds.

## Author contributions

**Conceptualization:** Do Kyun Kim.

**Data curation:** Jin Hee Jung, Ikwan Chang.

**Formal analysis:** Dongbum Suh, Jin Hee Lee.

**Methodology:** Ikwan Chang, Jae Yun Jung.

**Supervision:** Jin Hee Jung, Young Ho Kwak.

**Validation:** Jae Yun Jung.

**Writing – original draft:** Dongbum Suh.

**Writing – review & editing:** Jin Hee Jung, Ikwan Chang, Jin Hee Lee, Young Ho Kwak, Do Kyun Kim.

Do Kyun Kim orcid: 0000-0002-6144-302X.
